# Patterns of Infections among Extremely Preterm Infants

**DOI:** 10.3390/jcm12072703

**Published:** 2023-04-04

**Authors:** Krystle Perez, Mihai Puia-Dumitrescu, Bryan A. Comstock, Thomas R. Wood, Dennis E. Mayock, Patrick J. Heagerty, Sandra E. Juul, on behalf of the PENUT Consortium

**Affiliations:** 1 Division of Neonatology, Department of Pediatrics, University of Washington School of Medicine, Seattle, WA 98195, USA; 2 Department of Biostatistics, University of Washington, Seattle, WA 98195, USA

**Keywords:** extremely preterm infants, neonatal infections, exposure to antibiotics, sepsis

## Abstract

Infections remain a leading cause of neonatal death, especially among the extremely preterm infants. To evaluate the incidence, pathogenesis, and in-hospital outcomes associated with sepsis among hospitalized extremely preterm infants born at 24–0/7 to 27–6/7 weeks of gestation, we designed a post hoc analysis of data collected prospectively during the Preterm Epo Neuroprotection (PENUT) Trial, NCT #01378273. We analyzed culture positive infection data, as well as type and duration of antibiotic course and described their association with in-hospital morbidities and mortality. Of 936 included infants, 229 (24%) had at least one positive blood culture during their hospitalization. Early onset sepsis (EOS, ≤3 days after birth) occurred in 6% of the infants, with Coagulase negative Staphylococci (CoNS) and Escherichia Coli the most frequent pathogens. Late onset sepsis (LOS, >day 3) occurred in 20% of the infants. Nearly all infants were treated with antibiotics for presumed sepsis at least once during their hospitalization. The risk of confirmed or presumed EOS was lower with increasing birthweight. Confirmed EOS had no significant association with in-hospital outcomes or death while LOS was associated with increased risk of necrotizing enterocolitis and death. Extremely premature infants with presumed sepsis as compared to culture positive sepsis had lower rates of morbidities. In conclusion, the use of antibiotics for presumed sepsis remains much higher than confirmed infection rates. Ongoing work exploring antibiotic stewardship and presumed, culture-negative sepsis in extremely preterm infants is needed.

## 1. Introduction

Sepsis remains a leading cause of neonatal death, especially among those born prematurely [1,2]. Infection has been implicated as a contributor to more than 10% of deaths among extremely preterm (EP) infants [3]. Additionally, infection survivors often have a longer length of stay, higher risk of morbidities including bronchopulmonary dysplasia (BPD), intraventricular hemorrhage (IVH), retinopathy of prematurity (ROP) [4], and worse neurodevelopmental outcomes following discharge from the hospital [5]. 

The pathophysiology of early versus late onset sepsis (LOS) is distinct. Early onset sepsis (EOS) occurs within the first 3 days after birth and is most often related to intrapartum, vertical transmission of bacteria. LOS occurs beyond the first 3 days after birth after neonates are colonized with bacteria [6]. Confirmation of sepsis requires isolation of an organism in a blood culture, though in the context of significant clinical signs infants may also carry the diagnosis of culture-negative or presumed sepsis. It is not well described whether EOS versus LOS or confirmed versus presumed sepsis have differential impacts on in-hospital outcomes. 

EP infants are at increased risk of LOS due to immaturity of the immune system, high prevalence of central lines, and high need for invasive procedures. While historically *Escherichia coli* (*E. coli*) and Group B Streptococcus (GBS) are the most frequent pathogens associated with EOS [7,8,9], this pattern may be changing in the setting of routine GBS prophylaxis among laboring, GBS-colonized women regardless of infant gestational age. To determine the incidence of confirmed and presumed sepsis, causative bacteria and use of antibiotics among a contemporary cohort of EP infants, we used the Preterm Erythropoietin Neuroprotection (PENUT) Trial’s database. The PENUT Trial prospectively enrolled and randomized 941 EP infants at 19 sites, 30 neonatal intensive care units (NICUs) and 13 states across the U.S. We also evaluated candidate patient factors that were associated with increased risk of infection, and whether culture-positive infections or presumed infections were associated with an increased risk of subsequent adverse events. 

## 2. Materials and Methods

We conducted a post hoc analysis of data collected prospectively during the PENUT Trial, NCT #01378273. All infants enrolled in the PENUT trial were eligible for this analysis of infections [10]. The PENUT Trial was a phase III randomized placebo-controlled double blinded trial in which EP infants born at 24 0/7 to 27 6/7 weeks’ gestation were randomized to receive placebo or erythropoietin (Epo) from the first day after birth to 32 weeks post-menstrual age (PMA). The trial was approved by the institutional review board at each participating site and parent consent obtained. Infants were enrolled between December 2013 and September 2016. Infants either received 1000 U/kg Epo or placebo intravenously for 6 doses, followed by subcutaneous injections of 400 U/kg Epo or sham injections three times a week through 32 weeks PMA. The study protocol excluded inborn infants with a diagnosis of congenital brain malformations or known congenital infection such as toxoplasmosis, cytomegalovirus, rubella or syphilis at the time of enrollment. 

### 2.1. Participant Characteristics

The following maternal characteristics were collected: race and ethnicity, education, prenatal care, prolonged rupture of membranes, chorioamnionitis, hypertension (PIH, pre-eclampsia, eclampsia), obesity, preterm labor, antenatal steroids, antibiotic exposure, antenatal magnesium therapy, multiple gestation, and drug or alcohol use. Race and ethnicity were self-reported. Baseline infant characteristics collected were mode of delivery, sex, gestational age, birth weight including small for gestational age status, Apgar score at 5 min, and whether delayed cord clamping (>30 s) was performed. 

### 2.2. Definition of Infection

All clinically identified confirmed positive blood cultures were prospectively recorded as a part of the PENUT Trial protocol with cultured organisms (bacterial, fungal or viral) identified by name, sample date, and Gram-stain (negative, positive). All antibiotic treatment start and stop dates were recorded as a part of the study protocol, though indication for antibiotic use was unavailable. Presumed infections were defined as receipt of antibiotic treatment continuously for 5 or more days in the absence of a positive blood culture. Given this definition, infants who died within the first 5 days after birth were excluded from analyses of presumed infections. Confirmed and presumed infections identified within the first 120 days were included in the analyses. Infections identified on day 0 through 3 were categorized as EOS and those identified after the third day were categorized as LOS for this analysis. Only blood cultures were included for this analysis.

### 2.3. Severe Adverse Events

Severe adverse events included severe ROP (defined as requiring treatment with laser photocoagulation, cryotherapy or bevacizumab), severe IVH (defined as Grade III or IV, either unilateral or bilateral, according to Papile staging) [11], severe BPD (defined as requiring nasal cannula or higher levels of respiratory support at 36 weeks PMA), Bell’s stage 2b or 3 necrotizing enterocolitis (NEC) [12], and mortality. For each adverse event, the date of identification was recorded.

### 2.4. Statistical Analysis

We used simple descriptive statistics to tabulate types and timing of confirmed infections and antibiotic treatment. For confirmed and presumed infections, infants were descriptively classified into one of four categories based on their infection(s) timing: (1) No infections; (2) EOS only; (3) LOS only, and (4) Both EOS and LOS. Baseline maternal and infant characteristics were summarized using frequency counts (%) by infection timing category, and Fisher exact tests were used to screen for differences in infection rates between levels of each predictor. We aimed to evaluate the independent contributions of candidate baseline maternal and infant characteristics as predictors of EOS and LOS presumed or confirmed infections. Due to intermittent missing data across the set of predictors, we first conducted a multiple imputation analysis (mice R package [13]; m = 10 imputations) to impute values for missing predictors. To appropriately account for potential correlation of outcomes for same-birth siblings, combinations of variables were then selected with generalized estimating equation (GEE) models for EOS and LOS infection status (separately for confirmed and presumed) based upon improvement in the working independence Akaike Information Criteria (AIC) metric [14]. AIC is an estimator of predictive performance of a given model, balancing the tradeoff between goodness-of-fit and complexity, and is applicable to non-nested models. With the final set of selected variables, we used GEE models with robust standard errors for statistical inference. 

Associations between presumed or confirmed infections and severe adverse events (ROP, IVH, BPD, NEC, death) were examined using Cox proportional hazards models, with adjustment for treatment with Epo, gestational age, and variables found to predict presumed or confirmed infections, respectively. Presumed or confirmed infection status was coded as time-varying covariates and included separately in Cox models for each adverse event, using time of the earliest identified presumed or confirmed infection to classify into EOS or LOS. Thus, infections determined by blood culture or initiation of antibiotics after a severe adverse event were censored and not included in the analyses.

All analyses were conducted using the R statistical package (version 4.0.2, Foundation for Statistical Computing, Vienna, Austria, https://www.R-project.org accessed on 22 June 2000) and no corrections were made for multiple statistical tests.

## 3. Results

**Figure 1:**
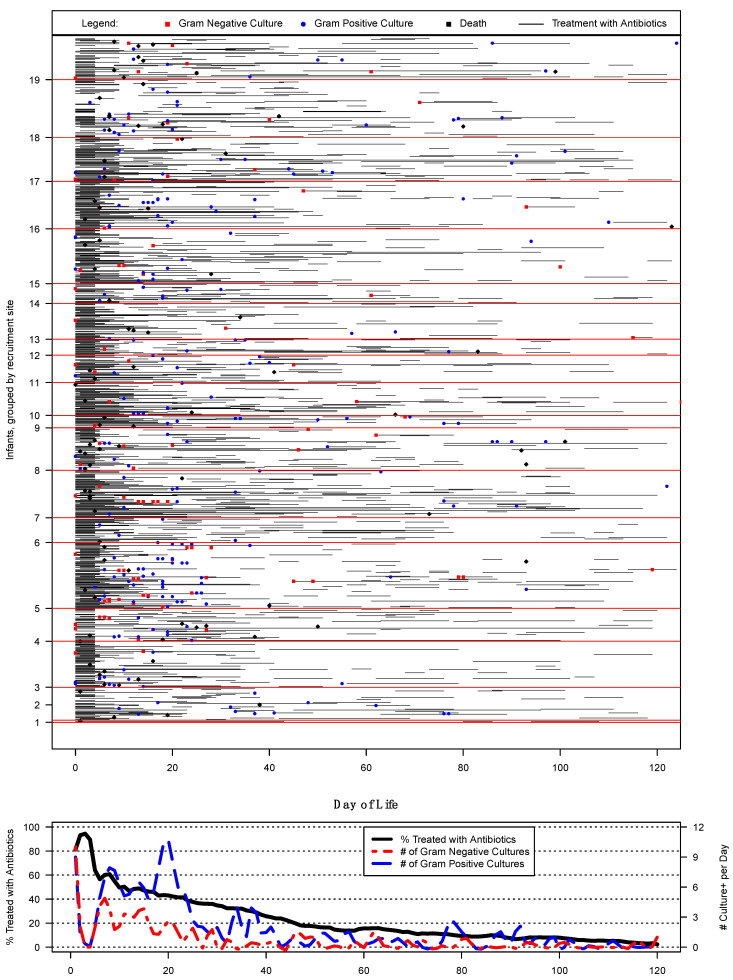
Swimmer plot of all infants enrolled at each site; a black horizontal line represents one antibiotic treatment course (with length of treatment represented by the length of the horizontal line) by days of life along the x-axis. Colors and symbols denote the status of a positive blood culture or death according to the figure legend embedded above.

Of the 936 EP infants included in this study, 230 (25%) had one or more positive blood culture(s) during their hospitalization. Among the 909 infants eligible to have had at least 5 days of antibiotics, 798 (88%) had at least one episode of presumed sepsis. Only 64/909 (7%) infants did not have a diagnosis of confirmed or presumed sepsis during their hospital stay. No difference was noted between Epo-treated and placebo groups in either the type or incidence of infection (Table 1). There was considerable site variation in the incidence and type of infection with range from 0 to 12% for confirmed EOS and 10% to 33% for confirmed LOS among the 19 sites (Figure 1). In this cohort, the use of antibiotics was extremely common across all sites with a median (IQR) percentage of infants exposed of 88% (86%, 92%). 

### 3.1. Confirmed Infections

Demographics by timing of confirmed sepsis can be found in Table 1. Of the 936 infants, 59 (6%) had EOS. Of the 60 bacterial species identified, the most commonly responsible pathogens for EOS in this cohort were CoNS (*n* = 23), followed by *E. coli* (*n* = 13), and Pseudomonas species (*n* = 3) (Supplemental Table S1). GBS accounted for 2 cases of EOS. LOS occurred in 190 (20%) of the 915 infants surviving past 3 days. The most reported pathogens for LOS were CoNS (*n* = 117), other Staphylococcus species (*n* = 39, including Methicillin Resistant *Staphylococcus aureus*). Klebsiella, *E. coli* and Enterococcus species comprised equal portions (*n* = 22, 21, and 20, respectively). There were 19 (2%) infants who had both confirmed EOS and LOS. Among the 230 infants with confirmed sepsis, 161 (70%) had a one, 42 (18%) had two, and 27 (12%) had three or more confirmed infection episodes during their hospitalization.

### 3.2. Presumed Infections

There were 909 infants eligible for the diagnosis of presumed sepsis due to the requirement to survive for 5 days (to have at least 5 days of consecutive antibiotic exposure in the absence of a positive blood culture). Demographics by timing of presumed sepsis can be found in Table 2. Of the 909 infants eligible for a diagnosis of presumed sepsis, 798 (88%) had at least one episode of presumed sepsis during their hospital stay. Based upon the initial day of antibiotics, 374 (41%) of infants had presumed sepsis diagnosed within the first three days, 107 (12%) beyond three days, and 317 (35%) had both. Among the 798 infants with presumed sepsis, 443 (56%) had one, 232 (29%) had two, and 123 (15%) had three or more presumed infections.

### 3.3. Infection Risk Factors

**Figure 2:**
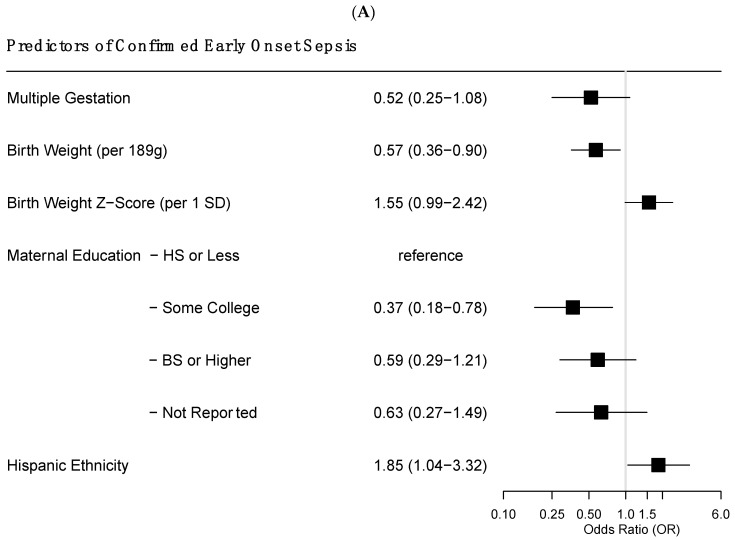

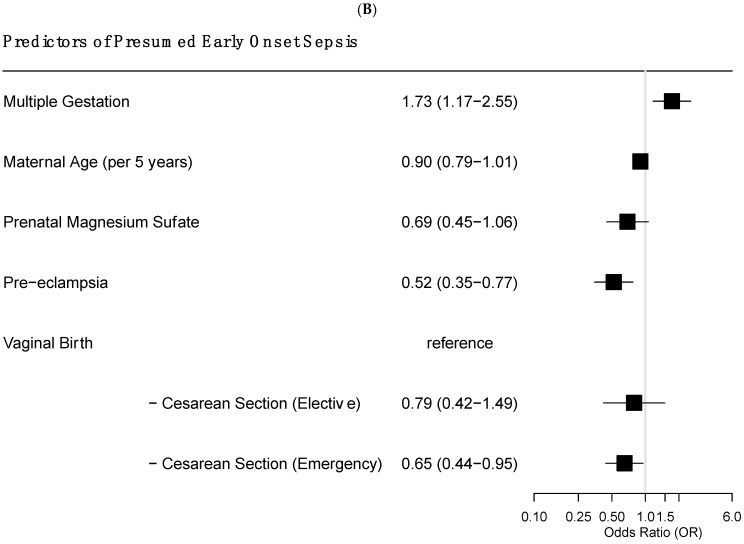
This is a figure. Forest plot of predictors of confirmed (**A**) and presumed (**B**) early onset sepsis. In this independent, multivariable association, significant differences were noted for birthweight, maternal education, and Hispanic ethnicity for confirmed early onset sepsis (**A**); significant differences were noted for multiple gestation, pre-eclampsia and emergent cesarean section for presumed early onset sepsis (**B**).

Predictors of confirmed EOS included birthweight, maternal education, and Hispanic ethnicity (Figure 2A). Specifically, infants of higher birth weight had a lower risk of EOS (RR = 0.57 per standard deviation (189 g) increase; 95% CI = 0.36–0.90). Infants whose maternal education was greater than high school tended to have lower risks of EOS, with some college education associated with the least risk of confirmed EOS (RR = 0.37; 95% CI 0.18–0.78). Infants of mothers that identified as Hispanic ethnicity were more likely to have EOS compared to non-Hispanic mothers (RR = 1.85; 95% CI = 1.04–3.32). 

Infants without presumed sepsis tended to have higher rates of emergent Cesarean section and maternal preeclampsia when compared to infants with at least one episode of presumed sepsis (Figure 2B). 

### 3.4. Antibiotic Therapy

Most infants (*n* = 691, 74%) were exposed to antibiotics in the first 72 h after birth (Figure 1). Supplemental Table S2 summarizes the antibiotics used in this cohort. The most used antibiotics for EOS or presumed sepsis in the first 3 days were ampicillin (47%) followed by gentamicin (43%). Nearly half of infants (*n* = 424, 47%) experienced presumed sepsis beyond 3 days with the most used antibiotics being gentamicin (27%) and vancomycin (25%). The median (IQR) continuous duration of antibiotics for presumed sepsis was 9 (5, 13) days. The median (IQR) duration of antibiotics for confirmed sepsis was 18 (12, 29) days with courses slightly longer for EOS [median (IQR): 26 (17,38) days] compared to LOS [median (IQR): 16 (10,21) days].

### 3.5. In-Hospital Outcomes

**Figure 3:**
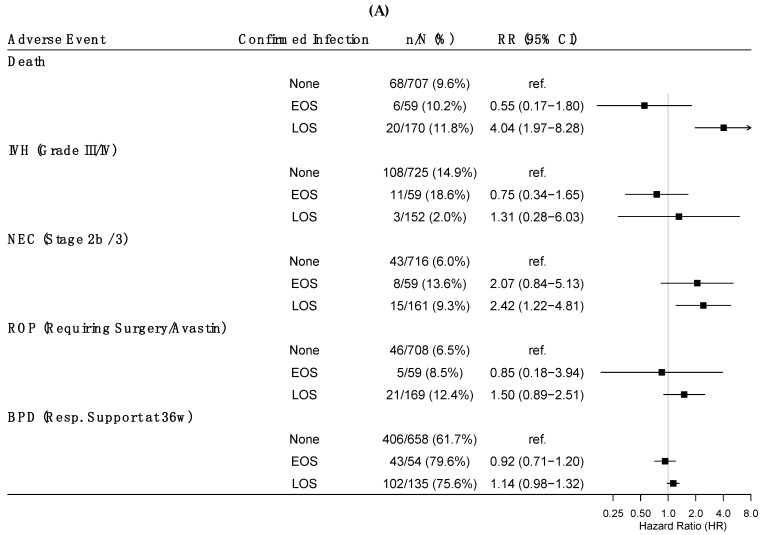

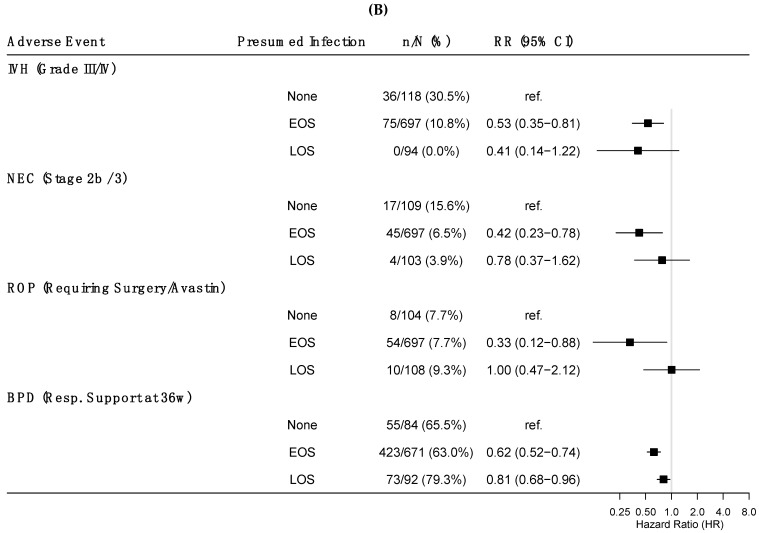
Forest plot of predictors of safety outcomes by confirmed (**A**) and presumed (**B**) early and late onset sepsis. Significant differences were noted for confirmed late onset sepsis and risk of death and necrotizing enterocolitis (**A**). Significant differences were noted for presumed early onset sepsis across all safety outcomes and for presumed late onset sepsis only for risk of bronchopulmonary dysplasia (**B**). EOS, Early onset sepsis; LOS, Late onset sepsis; IVH, intraventricular hemorrhage; BPD, bronchopulmonary dysplasia; NEC, necrotizing enterocolitis; ROP, retinopathy of prematurity.

Confirmed EOS was not significantly associated with death, severe IVH, NEC, severe ROP or BPD in this cohort (Figure 3A). Confirmed LOS was associated with increased risk of subsequent death (RR = 4.04; 95% CI = 1.97–8.28) and NEC (RR = 2.42; 95% CI = 1.22–4.81). Presumed EOS was associated with lower risk for all in hospital outcomes, while presumed LOS was associated with lower risk of BPD (RR = 0.81; 95% CI = 0.68–0.96) (Figure 3B). 

Of the 64 EP infants without a diagnosis of confirmed or presumed sepsis, 63% had BPD, 38% died, 17% had IVH, 11% had NEC, 4.7% were diagnosed with ROP.

### 3.6. Figures and Tables

The associated graph below the swimmer plot represents the *percentage* of infants treated with antibiotics (black line) relative to the absolute numbers of positive Gram-negative cultures (dotted red line) and Gram-positive blood cultures (blue line) by days of life.

## 4. Discussion

In our post hoc analysis of this contemporary cohort of EPs, most infants were diagnosed with a confirmed and/or presumed infection at least once during their hospitalization. Specifically, 24% of infants had a positive blood culture and confirmed sepsis at some point during their initial hospital stay. Approximately 6% of the infants in this cohort had confirmed EOS, which is higher than has been reported in larger cohorts of very low birthweight infants ≤ 1500 g with an estimated incidence of EOS of 1–2% [7,8,15,16,17]. The somewhat higher incidence in our cohort may be related to the degree of extreme prematurity, though incidence of EOS in a similarly modern cohort from the NICHD Neonatal Research Network cited just under 2% incidence of EOS among 22- to 28-week infants [15]. The higher incidence of sepsis in our cohort could be explained by the inability to determine whether some early positive cultures were due to contamination. Specifically, 23 infants with EOS were culture positive for CoNS, and excluding these from the analysis by presuming contamination, incidence of EOS in our cohort would drop to 3.6%. 

LOS affected one in five EPs in our cohort, which is similar to the 2002 NICHD [18] report among very low birthweight infants and somewhat less than the one-third reported in 2022 among very low birthweight infants born in the Netherlands [19]. Although the burden of LOS was relatively high, the burden of presumed sepsis was even higher. Nearly 90% of EPs in this cohort had at least one course of ≥5 days of antibiotics without a positive blood culture during their hospitalization. Interestingly, even with inter-site variability in incidence of EOS (0 to 12%) and LOS (10 to 33%), the incidence of presumed sepsis seemed relatively consistent across the 19 sites (range 86 to 92%). The high occurrence of presumed sepsis, or culture negative sepsis, is consistent with prior reports [20,21,22]. Unfortunately, the incidence of prolonged antibiotic exposure throughout an EP’s hospitalization has not significantly changed despite increasing awareness and efforts to improve antibiotic stewardship. 

When assessing the types of bacteria identified, we found that most common pathogens responsible for EOS were CoNS and *E. coli* with Pseudomonas and GBS comprising a minority. The low prevalence of GBS EOS may reflect the guidelines informing universal GBS screening and treatment, even in the setting of preterm birth. Our findings are distinct from those previously published, with unknown differences in GBS prophylaxis differences among our cohort compared to cohorts previously published and similar occurrence of Pseudomonas and GBS [7,8,9,17]. Consistent with prior reports, we found that CoNS was also the primary pathogen of LOS, constituting over 60% of the cases, followed by other Staphylococcus species including methicillin resistant *Staphylococcus aureus*. Klebsiella, *E. coli* and Enterococcus had similar distributions as causal bacteria for LOS and, together, they comprised one-third of the cases of LOS [8,9,15,17]. 

The most frequent antibiotics used in our cohort align well with causative bacteria, though active considerations may be given to reducing broad-spectrum antibiotic use upon confirmation of Gram-staining given these causal bacteria. The most frequently utilized antibiotics, namely ampicillin and gentamicin for EOS and gentamicin and vancomycin for LOS, were also noted on a recent survey of NICUs across 38 European countries [23]. Unfortunately, a 2021 Cochrane analysis confirmed the lack of evidence for choice of antibiotics for EOS and LOS with no documented differences in mortality or outcomes with overall low-certainty of evidence, high-risk of bias, and underpowered randomized trials to-date [24,25]. Studies assessing ideal antibiotic regimens, especially for LOS in the NICU, are warranted. Additionally, we noted a relatively broad range of duration of antibiotic course across confirmed and presumed, EOS and LOS. We found slightly higher median duration of antibiotic days for presumed infection courses than expected. We also noted longer courses of antibiotics for the confirmed infections in this cohort with the median course nearly 3 weeks in duration. Similar to the exact antibiotic regimen itself, limited literature exists describing outcomes associated with shorter (7 to 10 day) courses versus longer (14 to 21 day) courses of antibiotics for confirmed sepsis, though there is some limited evidence exploring shorter courses may be just as effective among preterm infants 32 weeks and greater and/or 1500 g or greater [26,27,28]. 

Higher birthweight was associated with reduced risk of EOS and LOS. We found differential risk of EOS with maternal education and ethnicity in our cohort. Infants from mothers of lower educational attainment had higher risk of EOS, with a consistent decrease in the trend with some college education appearing protective. Thus, it remains to be determined if increased risk relative to these maternal characteristics is related to differential microbiota, differential prenatal interventions, or differences in pre- or postnatal care that may further affect risk. Exploration of whether differences in care exist is potentially an important next step in determining drivers of this disparity in EOS risk. 

Confirmed EOS did not appear to have a significant association with subsequent in-hospital outcomes. Presumably, this may have been informed by the common practice of early screening and empiric antibiotics whilst awaiting culture results after birth for a majority of EPs, leading to earlier and immediate antibiotic therapy. The commonality of early antibiotic administration is best depicted in Figure 1, with the near solid column of black representing initial antibiotic use. Confirmed LOS, on the other hand, was associated with higher rates of death, which is partly attributable to our finding that approximately one-third of these cases did not have an associated antibiotic course. Screening and treatment of LOS is particularly challenging in neonatology, noting the signs and symptoms of sepsis may be similar to many other complications of prematurity including lung disease, feeding difficulties, and thermoregulatory challenges. Interestingly, presumed sepsis, especially within the first 3 days, was protective against all in-hospital outcomes analyzed. It is worth mentioning, however, that most infants were exposed to longer courses of antibiotics in our cohort. In contrast, death and BPD occurred less among the cohort with presumed LOS. This finding may be attributable to the reduction of inflammatory markers contributing to these diagnoses, though interpretation is made challenging by the overwhelming prevalence of presumed sepsis. 

The strengths of our study included the multi-site nature, capturing a broad range of both academic and non-academic centers with various practices. Furthermore, our retrospective review of the PENUT cohort provides a large cohort of EP infants where analysis of relatively infrequent events, such as EOS, can be better captured and described. 

Our study also has some limitations. Given the retrospective nature of the analysis, causality cannot be inferred. Additionally, we are unable to comment on incidence of sepsis per live births noting the retrospective analysis and requirement for eligibility into the original PENUT Trial. We were unable to distinguish suspected contaminants versus true positive cultures as well, which may have over captured culture-positive sepsis in our cohort. Specifically, we note a high CoNS sepsis rate, presumable with some of those blood cultures demonstrating CoNS likely contaminant. There were no biomarkers available to analyze, and available data lacked granularity on clinical indications for continuing antibiotics among those we categorized as presumed sepsis. This is particularly notable having excluded analysis of tracheal and urine cultures within the cohort, noting challenges in the correlation of symptoms versus colonization. CSF cultures were rarely reported in the dataset, leading to their exclusion to the current analysis. As such, presumed sepsis may also have been related to a confirmed infection elsewhere with a negative blood culture. 

## 5. Conclusions

The use of antibiotics for presumed sepsis remains much higher than confirmed infection rates. Ongoing work exploring ways to promote antibiotic stewardship while acknowledging the common diagnosis of presumed, culture-negative sepsis in EPs is needed.

## Figures and Tables

**Table 1:** Demographics by timing of confirmed sepsis.

Patient Characteristic	No Infection	Confirmed Sepsis			*p* Value
		EOS only	LOS only	EOS and LOS	
N (936)	706 (75%)	40 (4.3%)	171 (18%)	19 (2.0%)	
Treatment Group					0.65
Placebo	340 (74%)	22 (4.8%)	87 (19%)	11 (2.4%)	
Epo	366 (77%)	18 (3.8%)	84 (18%)	8 (1.7%)	
Gestational Age at Birth					<0.001
24w	150 (65%)	17 (7.3%)	59 (25%)	6 (2.6%)	
25w	175 (71%)	9 (3.7%)	54 (22%)	7 (2.9%)	
26w	174 (79%)	8 (3.6%)	35 (16%)	4 (1.8%)	
27w	207 (87%)	6 (2.5%)	23 (9.7%)	2 (0.8%)	
Birth weight	810 (190)	781 (171)	768 (182)	735 (218)	0.02
Sex					0.60
Male	361 (74%)	20 (4.1%)	95 (19%)	12 (2.5%)	
Female	345 (77%)	20 (4.5%)	76 (17%)	7 (1.6%)	
Multiple gestation					0.25
Singleton	516 (74%)	36 (5.2%)	127 (18%)	14 (2.0%)	
Multiple	190 (78%)	4 (1.6%)	44 (18%)	5 (2.1%)	
Maternal age, mean (SD)	28.9 (6.2)	29.3 (6.7)	29.6 (6.3)	26.6 (5.0)	0.63
Maternal education					0.05
HS or less	223 (73%)	19 (6.2%)	54 (18%)	11 (3.6%)	
Some college	211 (74%)	6 (2.1%)	64 (22%)	4 (1.4%)	
BS or greater	183 (79%)	10 (4.3%)	37 (16%)	2 (0.9%)	
Not reported	89 (79%)	5 (4.5%)	16 (14%)	2 (1.8%)	
Maternal Race					0.66
Black	183 (76%)	10 (4.2%)	40 (17%)	7 (2.9%)	
Other	42 (75%)	2 (3.6%)	10 (18%)	2 (3.6%)	
White	463 (76%)	24 (3.9%)	116 (19%)	8 (1.3%)	
Maternal Ethnicity					0.05
Non-Hispanic/Not Reported	559 (76%)	26 (3.5%)	139 (19%)	12 (1.6%)	
Hispanic	147 (74%)	14 (7.0%)	32 (16%)	7 (3.5%)	
Chorioamnionitis					0.33
No	619 (76%)	32 (3.9%)	145 (18%)	18 (2.0%)	
Yes	87 (71%)	8 (6.6%)	26 (21%)	1 (0.8%)	
Prenatal steroids					0.03
No	82 (87%)	3 (3.2%)	8 (8.5%)	1 (1.1%)	
Yes	624 (74%)	37 (4.4%)	163 (19%)	18 (2.1%)	
Apgar score @ 5 min					0.22
<5	132 (70%)	10 (5.2%)	42 (22%)	5 (2.6%)	
≥5	572 (77%)	30 (4.0%)	128 (17%)	14 (1.9%)	
Delayed cord clamping					0.73
No, Unknown	458 (74%)	27 (4.4%)	119 (19%)	13 (2.1%)	
Yes	247 (78%)	13 (4.1%)	52 (16%)	6 (1.9%)	
Delivery method					0.56
Cesarean-section	64 (82%)	3 (3.8%)	10 (13%)	1 (1.3%)	
Emergency Cesarean-section	434 (76%)	22 (3.8%)	103 (18%)	14 (2.4%)	
Vaginal	208 (73%)	15 (5.3%)	58 (20%)	4 (1.4%)	

**Table 2:** Demographics by timing of presumed sepsis status.

Patient Characteristic	No Presumed Sepsis	Presumed Sepsis			*p* Value
		EOS only	LOS only	EOS and LOS	
*n* (909)	102 (11%)	351 (39%)	110 (12%)	346 (38%)	
Treatment Group					0.45
Placebo	49 (11%)	167 (37%)	62 (13%)	171 (38%)	
Epo	53 (12%)	184 (40%)	48 (10%)	175 (38%)	
Gestational Age at Birth					0.16
24w	21 (9.7%)	77 (35%)	30 (14%)	89 (41%)	
25w	21 (8.8%)	89 (37%)	29 (12%)	100 (42%)	
26w	34 (16%)	80 (37%)	28 (13%)	77 (35%)	
27w	26 (11%)	105 (45%)	23 (9.8%)	80 (34%)	
Birth weight	807 (202)	822 (190)	758 (197)	795 (177)	0.10
Sex					0.75
Male	48 (10%)	186 (40%)	58 (12%)	177 (38%)	
Female	54 (12%)	165 (38%)	52 (12%)	169 (38%)	
Multiple gestation					0.05
Singleton	90 (13%)	275 (38%)	88 (13%)	224 (37%)	
Multiple	21 (8.6%)	99 (41%)	19 (8.2%)	93 (42%)	
Maternal age, mean (SD)	30.2 (5.9)	28.6 (6.1)	29.3 (5.9)	28.8 (6.4)	0.32
Maternal education					0.22
HS or less	31 (10%)	120 (40%)	42 (14%)	107 (36%)	
Some college	34 (12%)	110 (39%)	28 (10%)	107 (38%)	
BS or greater	25 (11%)	81 (36%)	35 (16%)	84 (37%)	
Not reported	12 (11%)	40 (38%)	5 (4.8%)	48 (46%)	
Maternal Race					0.39
Black	25 (11%)	90 (38%)	24 (10%)	97 (41%)	
Other	7 (13%)	15 (27%)	10 (18%)	23 (42%)	
White	66 (11%)	240 (41%)	70 (12%)	214 (36%)	
Maternal Ethnicity					0.93
Non-Hispanic/Not Reported	79 (11%)	280 (39%)	88 (12%)	271 (38%)	
Hispanic	23 (12%)	71 (37%)	22 (12%)	75 (39%)	
Chorioamnionitis					0.16
No	91 (12%)	294 (37%)	100 (13%)	305 (39%)	
Yes	11 (9.2%)	57 (48%)	10 (8.4%)	41 (34%)	
Pre-eclampsia					<0.001
No	71 (9.3%)	297 (39%)	83 (11%)	310 (41%)	
Yes	31 (21%)	54 (36%)	27 (18%)	36 (24%)	
Prenatal steroids					0.76
No	8 (9.1%)	33 (38%)	9 (10%)	38 (43%)	
Yes	94 (11%)	318 (39%)	101 (12%)	308 (38%)	
Apgar score @ 5 min					0.43
<5	24 (14%)	61 (35%)	19 (11%)	71 (41%)	
≥5	78 (11%)	290 (40%)	91 (12%)	272 (37%)	
Delayed cord clamping					0.95
No, Unknown	71 (12%)	249 (42%)	70 (12%)	206 (35%)	
Yes	40 (13%)	125 (40%)	37 (12%)	110 (35%)	
Delivery method					0.008
Elective Cesarean-section	8 (10%)	32 (42%)	9 (12%)	29 (36%)	
Emergency Cesarean-section	75 (14%)	216 (39%)	74 (13%)	189 (34%)	
Vaginal	19 (6.8%)	103 (37%)	27 (9.7%)	129 (46%)	

## Data Availability

The data used to support the findings in this study are available upon reasonable request.

## Supplementary Materials

The following supporting information can be downloaded at: https://www.mdpi.com/article/10.3390/jcm12072703/s1, Table S1: Blood cultures by Gram-stain and timing of infection; Table S2: Antibiotic medication names by infection timing and status.
