# Supplementary material for: Patterns of Infections among Extremely Preterm Infants
Source: J Clin Med. 2023 Apr 4;12(7):2703. doi: 10.3390/jcm12072703 (PMC10095151; doi:10.3390/jcm12072703)
Supplement: Supplementary file 1 [file jcm-12-02703-s001.zip › Supplemental Table S1.pdf]

Supplemental Table S1: Blood cultures by gram stain and timing of infection.

	Confirmed Infection	
	Early	Late
N	62	285
Blood Cultures		
Gram Negative	25	83
Gram Positive	37	202
Infection		
Coagulase Negative Staphylococci	24	119
Coagulase Positive Staphylococci, including Staphylococci Aureus	5	42
Klebsiella	1	22
E. coli	14	21
Enterococcus	1	19
Serratia	2	12
Pseudomonas	3	9
Group B Strep	2	9
Enterobacter	1	7
Other	11	25
