# Supplementary material for: Patterns of Infections among Extremely Preterm Infants
Source: J Clin Med. 2023 Apr 4;12(7):2703. doi: 10.3390/jcm12072703 (PMC10095151; doi:10.3390/jcm12072703)
Supplement: Supplementary file 1 [file jcm-12-02703-s001.zip › Supplemental Table S2.pdf]

Supplemental Table S2. Antibiotic medication names by infection timing and status.

	Confirmed Infection		Presumed Infection	
	EOS	LOS	EOS	LOS
N	134	405	1,445	1,246
Gentamicin	55	59	621	345
Ampicillin	59	15	684	93
Vancomycin	1	71	4	316
Cephalosporin	2	32	15	138
Antifungal	11	12	96	60
Piperacillin-Tazobactam	1	15	0	82
Oxacillin	0	13	2	66
Other	2	24	23	146
No qualifying antibiotic given	3	164	0	0
